# Isolated Exercise-Induced Pulmonary Hypertension Associates with Higher Cardiovascular Risk in Scleroderma Patients

**DOI:** 10.3390/jcm9061910

**Published:** 2020-06-18

**Authors:** Rosalinda Madonna, Riccardo Morganti, Francesco Radico, Piergiusto Vitulli, Marco Mascellanti, Paolo Amerio, Raffaele De Caterina

**Affiliations:** 1 Institute of Cardiology, University of Pisa, 56124 Pisa, Italy; rosalinda.madonna@unipi.it; 2 Institute of Epidemiology, University of Pisa, 56124 Pisa, Italy; r.morganti@ao-pisa.toscana.it; 3 Institute of Cardiology, G. d’Annunzio University—Chieti-Pescara, and SS. Annunziata Hospital, 56100 Chieti, Italy; francesco.radico@unich.it (F.R.); piergiusto@gmail.com (P.V.); 4 Cardiology Division, Ospedale Santo Spirito, 65127 Pescara, Italy; marco.mascellanti@ausl.pe.it; 5 Department of Medicine and Aging Sciences, Dermatology Clinic, G. d’Annunzio University—Chieti-Pescara, 56100 Chieti, Italy; p.amerio@unich.it; 6 Fondazione Villa Serena per la Ricerca, Città S. Angelo, 65013 Pescara, Italy

**Keywords:** exercise-induced pulmonary hypertension, scleroderma, cardio-pulmonary exercise test, echocardiography, cardiovascular risk

## Abstract

Background and Aim: Isolated exercise-induced pulmonary hypertension (ExPH) associates with cardiovascular (CV) events in patients with left heart disease. We investigated its prognostic significance in scleroderma patients at risk for pulmonary arterial hypertension (PAH). Methods: In 26 consecutive scleroderma female patients with either low (*n* = 13) or intermediate probability (*n* = 13) of pulmonary hypertension (PH) at rest, we evaluated, both at time 0 and 1 year, prognostic determinants of CV risk: onset or progression of heart failure/syncope; worsening of functional class; functional performance at the 6-minute walking test and at cardiopulmonary exercise test; right atrial area; and pericardial effusion. We assigned a severity score 1–3 to each prognostic determinant, derived an overall CV risk score, and its 0–1 year change. Isolated ExPH during the cardiopulmonary exercise test (CPET) was defined as absence of PH at rest, reduced peak VO_2_, VE/VCO_2_ >30 at anaerobic threshold, reduced O_2_ pulse, and ΔVO_2_/ΔW <9 mL/min/W. We then correlated ExPH at time 0 with clinical worsening (risk score increase >20% after 1 year). Results: ExPH was strongly associated with clinical worsening compared to patients without ExPH (*p* = 0.005). In patients without ExPH, none had > 20% increased CV risk score after 1 year. Conversely, about 50% of patients with ExPH had such an increase, suggesting a worsening of prognosis. Conclusions: Isolated ExPH associates with higher cardiovascular risk and thus clinical worsening in scleroderma patients. The assessment of ExPH by CPET can thus contribute to a better risk stratification and the planning of a more adequate follow-up.

## 1. Introduction

According to the recently updated European Society of Cardiology (ESC)/European Respiratory Society (ERS) PH guidelines, the term “pulmonary arterial hypertension” (PAH) describes a subpopulation of patients with pulmonary hypertension (PH) characterized hemodynamically by the presence of pre-capillary PH, defined as a mean pulmonary artery pressure ≥25 mmHg at rest, an expiratory pulmonary wedge pressure (PAWP) ≤15 mmHg, and a pulmonary vascular resistance >3 Wood units [1].

The first observation of PAH dates to 1891, when the German physician Ernst von Romberg described an autopsy case of “pulmonary vascular sclerosis”. A recent analysis from the Prospective Registry of Newly Initiated Therapies for Pulmonary Hypertension (COMPERA) [2] showed that the average age of first diagnosis of PAH is higher than that documented in older registries [3,4]. This indicates that the demographics of PAH is changing. Changes in PAH epidemiology now implies a late diagnosis, as the presence of co-morbidities in the elderly may mask the presence of PAH and make functional and haemodynamic impairment more severe. It also implies a worse natural history and a poorer response to therapy. PAH is a frequent complication of scleroderma (systemic sclerosis). Especially in scleroderma patients, such a diagnosis is usually delayed, and often made after the development of irreversible right heart dysfunction [2,3,4]. Early-on during their natural history, such patients may, however, develop an abnormal pulmonary hemodynamic response during exercise, due to impaired pulmonary vascular and right ventricular (RV) contractile reserve on effort, which can lead to impaired functional capacity and symptoms of effort intolerance [5]. The significance of exercise-induced pulmonary hypertension (ExPH) in patients at risk of developing PAH at rest, such as those with scleroderma, is unknown. In addition, the natural history of ExPH in these patients at risk of developing resting PAH appears variable, and longitudinal follow-up studies are needed.

We hypothesized that the non-invasive evaluation of pulmonary hemodynamics during exercise could be used for risk stratification in the follow-up of high-risk patients. We therefore investigated the prognostic significance of ExPH in a group of scleroderma patients.

## 2. Methods

### 2.1. Study Design and Data Collection

This was a prospective, observational, cohort study of 31 patients with scleroderma recruited from the Rheumatology Clinics of Chieti University Hospital (Chieti, Italy) and of the nearby Santo Spirito Hospital in Pescara, Italy. The study complied with the Helsinki Declaration, and informed consent was obtained from patients before each diagnostic test was done for clinical purposes. Patients eventually selected for the present report were later contacted and gave their approval for inclusion in the study protocol. Local investigators had full access to patient data and medical records.

**Figure 1:**
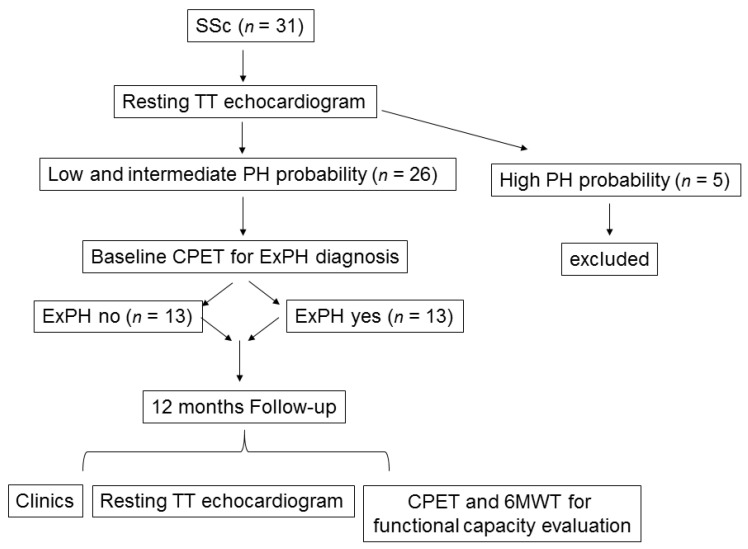
Study flowchart illustrating the study from enrollment to end of follow-up. Legend: CPET: cardiopulmonary exercise test; ExPH: exercise-induced pulmonary hypertension; PH: pulmonary hypertension; SSc: scleroderma; TT: transthoracic; 6MWT: six-minute walking test.

The study included 26 female patients with scleroderma. All enrolled patients underwent transthoracic echocardiography (TTE) prior to a cardiopulmonary exercise test (CPET). As shown in the flow-chart depicted in Figure 1, according to the echo evaluation of PH probability [1,6], we included patients with “low” PH probability (tricuspidal regurgitation velocity (TRV) ≤2.8 m/s or not measurable without additional PH signs, *n* = 13) and “intermediate” PH probability (TRV ≤2.8 m/s or not measurable, with additional PH signs; or TRV 2.9–3.4 m/s without additional PH signs, *n* = 13). We excluded patients with “high” PH probability (TRV 2.9–3.4 m/s with additional PH signs, or TRV >3.4 m/s, *n* = 5). Additional exclusion criteria were the presence of moderate-to-severe anemia (hemoglobin <10 g/dL); significant left heart disease at resting echocardiogram; history of venous thromboembolism, human immunodeficiency virus (HIV) infection, kidney disease, or chronic obstructive pulmonary disease that are all possible additional causes of PH; and moderate to severe tricuspid insufficiency (see below). Figure 1 depicts the echo evaluation of PH probability.

All patients received an intravenous infusion of Iloprost once a month (according to the scleroderma treatment protocol). All patients included in the study underwent CPET, which was performed according to the American College of Chest Physicians statement [7]. According to Berardinelli [8] and Wassermann [9], we defined ExPH as reduced oxygen uptake (peak VO_2_); minute ventilation relative to carbon dioxide production (VE/VCO_2_) >30 at anaerobic threshold; reduced O_2_ pulse <10mL/beat at peak exercise; and reduced amount of oxygen required at each load (ΔVO_2_/ΔW) <9 mL/min/W. The two groups of patients (with ExPH (*n* = 13), and without ExPH (*n* = 13) were compared at baseline (time 0) and after 1 year. We evaluated the following prognostic determinants of cardiovascular (CV) risk, according to the 2015 European Society of Cardiology (ESC)/European Respiratory Society (ERS) guidelines [1], in all patients at time 0 and after 1 year: (1) clinical signs of heart failure (absent/present); (2) syncope (absent/occasional/repeated); (3) worsening of World Health Organization Functional Class (WHO-FC) (I-II/III/IV); (4) deterioration of functional performance at CPET (peak VO_2_ >15, peak VO_2_ 11–15, peak VO_2_ <11; VE/VCO_2_ slope <36, VE/VCO_2_ slope 36–44.9, VE/VCO_2_ slope ≥45); (5) deterioration of functional performance at the 6-minute walking test (6MWT >440 meters, 165–440 meter, <165 meters); (6) echocardiographic parameters, including (a) right atrial (RA) area, estimated as < 18 cm^2^; 18–26 cm^2^; or >26 cm^2^; and (b) pericardial effusion, defined as absent, minimal, or moderate-to-severe. We assigned a severity score of 1–3 to each prognostic determinant (Table 1) and derived an overall CV risk score at 0 and 1 year.

### 2.2. Echocardiography

According to the recently updated ESC/ERS PH guidelines, we assessed the peak of tricuspid regurgitation velocity (TRV) to determine the echocardiographic probability of PH [1,6]. Additionally sought echocardiographic PH signs (classified as present if at least 2 of the 3 categories (A–C) were documented) were (A) Right ventricle/left ventricle basal diameter ratio >1.0; flattening of the interventricular septum (left ventricular eccentricity index >1.1 in systole and/or diastole); (B) Right ventricular acceleration time <105 ms and/or the presence of a mid-systolic notching; early diastolic pulmonary regurgitation velocity >2.2 m/s; pulmonary artery diameter >25 mm; (C) Inferior vena cava diameter >21 mm with decreased inspiratory collapse (<50% with a sniff or <20% with quiet inspiration); right atrial area (end-systole) >18 cm^2^ [1,6]. We assessed right ventricular function by measuring the tricuspid annular plane systolic excursion (TAPSE) and the fractional area change (FAC), according to the guidelines of the American Society of Echocardiography [10]. Since TAPSE and FAC can be load-dependent, we excluded from the study patients with moderate and severe tricuspid insufficiency (see above). We measured left ventricular ejection fraction (LVEF) using the biplane Simpson’s method [10]. We classified valvular disease from mild to moderate to severe according to current recommendations [10,11,12].

### 2.3. Cardiopulmonary Exercise Test

We performed a symptom-limited CPET on an electronically-braked cycle ergometer using a ramp-pattern increase in work rate. After calibration of the volumes and gas exchange analyzers, subjects breathed through a Rudolph mask connected with a two-way respiratory valve. We stopped the exercise test when one or more of the following criteria were present: achievement of predicted heart rate, fatigue, dyspnea, excessive arterial blood pressure increase (>230/130 mmHg), ≥2 mm ST-segment depression in at least two adjacent leads and/or angina. We considered achievement of the ischemic threshold as the onset of a 1 mm ST-segment depression in at least two adjacent leads [13]. We measured the anaerobic threshold with the V-slope method [14]. Peak oxygen uptake (VO_2_ peak) was the average oxygen uptake during the last 15 s of exercise, %VO_2_ max was the percent of VO_2_ peak predicted by anthropometric data, and % anaerobic threshold (AT) was the percentage of predicted AT. We defined O_2_ pulse as the oxygen uptake divided by the heart rate (HR), % O_2_ pulse as the percent of predicted O_2_ pulse [13,15], and VE/VCO_2_ and VE/VO_2_ were the slope of ventilation versus CO_2_ output and O_2_ uptake, respectively. The ΔVO_2_/ΔW slope was automatically calculated.

### 2.4. Statistical Analysis

We described categorical data by absolute frequency, and continuous data by mean and standard deviation (SD). To compare the qualitative and quantitative parameters with CPET diagnosis (ExPH no, ExPH yes), we used the chi square test and the t-test for independent samples (two-tailed), respectively. We calculated the “clinical worsening” variable based on the presence or absence of an increase in the CV risk score >20% after 1 year. To compare qualitative and quantitative parameters with the clinical worsening (no, yes), we applied the Fisher’s exact test and the t-test for independent samples (two-tailed), respectively. We set the statistical significance level at 0.05. We performed all analyses with the SPSS v.26 software.

## 3. Results

Out of the 26 patients included, 13 featured ExPH at baseline CPET, while the remaining 13 patients were negative. Demographics, clinical characteristics, echocardiographic, CPET, and 6MWT parameters at baseline (time 0) are shown in Table 2. At baseline (time 0) there were no significant differences between the two groups in terms of age, body surface area, heart rate, history of syncope, systolic blood pressure, history of high blood pressure, and concomitant medications (Table 2). None of the patients had any specific PAH treatments, and none received beta-blockers, aspirin, statins, oral anticoagulants, or digoxin. Three patients with ExPH and one patient without ExPH had a history of high blood pressure, while none had a history of other comorbidities such as diabetes mellitus, hypothyroidism, kidney disease, atrial fibrillation, lung disease, chronic obstructive pulmonary disease, venous thromboembolism, chronic thromboembolic disease, human immunodeficiency virus infection, coronary artery disease, myocardial revascularization, heart valve replacement, or congenital heart disease.

None of the patients were in WHO-FC IV or had a history of syncope. Four and nine patients with ExPH were in WHO-FC III and II, respectively, while most were in WHO-FC I (Table 2). The mean WHO-FC and 6MWT in the group of patients with ExPH were 2.3 ± 0.5 (range 2–3) and 406 ± 92 m respectively, while all patients without ExPH were in class I and walked on average 637 ± 57 m. During CPET, VO_2_ peak was 10.6 ± 4 mL/kg/min in patients with ExPH, and 23 ± 3 mL/kg/min in patients without ExPH (Table 2).

Echocardiography did not show dilatation of the right ventricle (RV) both in patients with ExPH (34 ± 0.8 mm in the apical 4-chamber view) and in patients without ExPH (31 ± 0.5 mm). There were no significant differences between the two groups in terms of left ventricular function assessed by ejection fraction (EF) or right ventricular (RV) function assessed by fractional area change (FAC). A significantly higher proportion of patients with ExPH had higher systolic pulmonary artery pressure (sPAP) (*p* = 0.0001), higher tricuspid regurgitant velocity (TRV) (*p* = 0.0001), higher right atrial area (*p* = 0.0001), and a slightly lower TAPSE (*p* = 0.03), compared to those in patients without ExPH (Table 2). No pericardial effusion was observed in any of the 26 patients. As for LV systolic function, LVEF was normal in both groups, with values of 62 ± 4% in patients with ExPH and 64 ± 3% in patients without ExPH.

The average duration of follow-up was 1 year (interquartile range IQR = 349–361; median = 363 days). During this time, no patients died. All 26 patients in the study underwent a CV risk score evaluation at baseline (time 0) and after 1 year. At the end of the follow-up, we assessed which patients had a deterioration greater than 20% of the CV risk score. Hence, we defined this increase as a clinical worsening. Comparisons of quantitative and qualitative clinical parameters with clinical worsening are shown in Table 3.

Stratified on the basis of presence/absence of ExPH, the two groups had a different progression of CV risk score at 1 year, with clinical worsening occurring more frequently in patients with a baseline diagnosis of ExPH. While in the group without ExPH, no patient had an increase in the CV risk score > 20% after 1 year, about 50% of patients with ExPH had such increase (Table 3). Thus, the presence of ExPH at baseline was associated with later clinical worsening compared to patients without ExPH (Table 3).

## 4. Discussion

In the present study, we assessed the existence of an association between ExPH assessed by CPET at baseline and the worsening of CV risk measured by the 1-year score in scleroderma patients with low or intermediate probability of PH with resting echocardiogram. We found that the presence of ExPH at baseline was associated with an increase (worsening) in the CV risk score >20% compared to the absence of ExPH, suggesting a worsening of prognosis in this group of patients with ExPH. Thus, the development of ExPH during a CPET performed at baseline was associated with increased CV risk at 1 year, as assessed by the risk score currently adopted by the ESC/ERS guidelines. These findings underscore the usefulness of the CPET in the CV risk evaluation of scleroderma patients.

Specifically, we found that ExPH at baseline was associated with a worse WHO-FC, less distance walked at the 6MWT, and a lower VO_2_ peak assessed at the same time. More importantly, however, we found that half of patients with ExPH have a worsened CV risk score at 1 year, as these patients had a greater increase in the CV risk score during the follow-up period compared to patients without ExPH. Thus, the worsening in CV risk was limited to the group with ExPH at baseline.

CV risk assessment in patients with scleroderma, at risk of developing PAH, has a major effect on the conduct of the follow-up and on therapeutic management. The 2015 ESC/ERS guidelines on the diagnosis and management of PH [1] indicate a series of prognostic determinants that allow for the 1-year CV risk estimate in patients with PAH at rest. In our study, the presence of ExPH was associated with a worsening of some of these prognostic determinants, mainly related to exercise and functional capacities, but added to the predictive power of those determinants at baseline. Thus, the development of ExPH at baseline can be considered an early marker of worse prognosis in patients with scleroderma at high risk of developing PAH.

According to clinical experience, some patients with scleroderma who are still oligosymptomatic do much better than others in terms of exercise capacity, WHO-FC, and therefore in quality of life. They may differ in their pulmonary vascular reserve, defined as the ability of the pulmonary circulation to adapt to a higher blood flow while maintaining low pressure during high exercise levels [16,17]. A high pulmonary vascular reserve is characterized by low pulmonary vascular resistance, a high pulmonary vascular distensibility, a high pulmonary capillary volume, and a high pulmonary diffusion capacity [16]. Thus, pulmonary vascular reserve is modulated by the functional state of the pulmonary circulation, and the greater the pulmonary vascular reserve, the lower the likelihood of progressing through risk classes. It is noteworthy to mention that the non-invasive estimate of the increase in PH during exercise, as described here, was obtained in patients who had not undergone any PAH-targeted treatment.

Recent advances in the medical management of PAH have led to the speculation that some treatments available for established PAH may also have benefit for patients with scleroderma and reduced pulmonary vascular reserve, before PH develops at rest. Currently, no agent used for PAH is approved for use in patients with scleroderma and ExPH, but still without PAH at rest, and to the best of our knowledge, no studies of this type are underway. Our findings, by showing that the non-invasive evaluation of ExPH may be useful in the follow-up evaluation of these patients, may be hypothesis-generating for the undertaking of such studies.

### Study Limitations

The limitations of our study include the evaluation of pulmonary hemodynamics without right heart catheterization. The non-invasive evaluation of PH during exercise can be difficult, as it can be hindered by various technical factors. These include the possible sympathetic reaction induced by the anxiety related to the need of breathing through a mask. Such evaluation is probably subject to low accuracy, and is at present not yet independently validated. Data on brain natriuretic peptide (BNP) and N-terminal pro brain natriuretic peptide (NT-proBNP) were not available for all patients in the present study, thus we could not investigate the correlation between these parameters and ExPH. We did not find a higher mortality in the ExPH group, probably due to the short follow-up and the small sample size.

## 5. Conclusions

ExPH is associated with higher cardiovascular risk and thus clinical worsening in scleroderma (SSc) patients. The assessment of ExPH by CPET can thus contribute to a better risk stratification of the patient and the planning of a more adequate follow-ups.

## Figures and Tables

**Table 1:** Score of cardiovascular risk.

Risk Factors	Score
**Clinical Signs of Heart Failure**	
Absent	1
Present	3
**Syncope**	
No	1
Occasional	2
Repeated	3
**Word Health Organization Functional Class**	
I, II	1
III	2
IV	3
**6-Minute Walking Test**	
>440 m	1
165–440 m	2
<165 m	3
**Peak VO_2_**	
>15 mL/min/Kg	1
11–15 mL/min/Kg	2
<11 mL/min/Kg	3
**VE/VCO_2_ Slope**	
<36	1
18–26 cm^2^	2
>26 cm^2^	3
**Pericardial Effusion**	
No	1
Minimal	2
Clearly present	3

Legend: Peak VO_2_: peak oxygen uptake; VE/VCO_2_ slope: slope of the ventilation vs. carbon dioxide relationship. The absence of a score equal to 2 in the heart failure category indicates the absence of an intermediate category.

**Table 2:** Baseline characteristics of the study population.

	All Patients	Without ExPH	With ExPH	*p*
	(*n* = 26)	(*n* = 13)	(*n* = 13)	
Age, y	69 ± 13	68 ± 12	69 ± 11	0.975
Height, cm	160 ± 5	157 ± 4	163 ± 5	**0.005**
Weight, kg	62 ± 10	62 ± 11	63 ± 10	0.837
BSA (m^2^)	1.6 ± 0.1	1.67 ± 0,1	1.58 ± 0,1	0.116
WHO-Functional Class, % (*n*)				
I/II	85 (22)	50 (13)	35 (9)	0.123
III	15 (4)	0 (0)	15 (4)	**0.01**
**Physiological Parameters**				
HR (beats/min)	69 ± 6	68 ± 4	69 ± 7	0.828
SBP (mmHg)	120 ± 3	119 ± 9	121 ± 10	0.574
**Co-morbidities, % (*n*)**				
Systemic arterial hypertension	15 (4)	4 (1)	11 (3)	0.22
6MWT (m)	522 ± 139	637 ± 56	406 ± 92	**0.0001**
**CPET**				
VO_2_ peak (% predicted value)	67 ± 22	85 ± 4	48 ± 16	**0.0001**
ΔVO_2_/ΔW (mL/min/W)	12 ± 4	16 ± 1	9 ± 3	**0.0001**
VE/VCO_2_	27 ± 13	15 ± 3	39 ± 8	**0.0001**
O_2_ pulse (mL/min)	9 ± 4	13 ± 2	6 ± 0.7	**0.0001**
**TT Echocardiogram**				
Right ventricular outflow doppler acceleration time (m/s)	128 ± 8	128 ± 9	128 ± 8	0.858
Inferior cava diameter (cm)	1.6 ± 0.3	1.6 ± 0.3	1.6 ± 0.2	0.866
Right atrial area (cm^2^)	15 ± 2	13 ± 1	16 ± 2	**0.0001**
sPAP (mmHg)	32 ± 14	20 ± 5	43 ± 8	**0.0001**
TRV (m/s)	2.3 ± 0.8	1.5 ± 0.3	3 ± 0.2	**0.0001**
TAPSE (mm)	23 ± 4	24 ± 3	21 ± 4	**0.031**
LVEF (%)	63 ± 4	64 ± 3	62 ± 4	0.121
**Conventional Therapies**				
ACEi/ARBs	15 (4)	4 (1)	11 (3)	0.277
Diuretics	8 (2)	0 (0)	8 (2)	0.141

Legend: BSA: body surface area; WHO: World Health Organization; HR: heart rate; SBP: systolic blood presssure; LVEF: left ventricle ejection fraction; TAPSE: tricuspid annular plane systolic excursion; TRV: tricuspid regurgitant velocity; sPAP: systolic pulmonary artery pressure; 6MWT: six-minute walking test; VO_2_ peak: peak oxygen uptake; DVO_2_/DW: amount of oxygen required at each load; VE/CO_2_ slope: minute ventilation relative to carbon dioxide production; ACEi: angiotensin converting enzyme inhibitors; ARBs: angiotensin II type 1 (AT1) receptor antagonists. *p*-values refer to two-tailed tests for differences between the groups with ExPH and without ExPH, *t*-test in case of quantitative variables, chi-square test for qualitative variables. Significant *p*-values are marked in bold.

**Table 3:** Comparison of Quantitative and Qualitative Demographic and Clinical Parameters According to the Presence or Absence of “Clinical Worsening” (Score Increase > 20% After 1 Year).

Parameters	Clinical Worsening		*p*
	No (*n* = 19)	Yes (*n* = 7)	
Age, y	69.7 (12.1)	66.7 (12.3)	0.579
BMI	24.2 (4.8)	25.2 (2.3)	0.607
**Physiological Parameters**			
HR (beats/min)	70 (5)	66 (9)	0.222
SBP (mmHg)	121 (10)	119 (9)	0.583
**TT Echocardiogram**			
TAPSE (mm)	23.2 (4)	22.1 (4)	0.574
RV FAC (%)	46.6 (6.0)	50.7 (4.2)	0.106
LVEF (%)	63 (4)	63 (4)	0.932
Mild aortic valve regurgitation			0.065
no	19	5	
yes	0	2	
**Score at Time 0**	9.4 (2.4)	9.3 (0.8)	0.884
**ExPH Diagnosis**			**0.005**
no	13	0	
yes	6	7	
**Conventional Therapies**			
ACEi/ARBs			0.287
no	17	5	
yes	2	2	
Diuretics			0.065
no	19	5	
yes	0	2	
Statins			0.999
no	18	7	
yes	1	0	

Legend: ExPH: exercise-induced pulmonary hypertension; BMI: body mass index; SBP: systolic blood pressure; TAPSE: tricuspid annular plane systolic escursion; RV FAC: right ventricular fractional area change; LV EF: left ventricular ejection fraction; ACEi: angiotensin converting enzyme inhibitors; ARBs: Angiotensin Receptor Blockers. Statistics: mean (SD) or frequency; bold indicates statistically significant numbers.
